# Thermally evaporated indium-free, transparent, flexible SnO_2_/AgPdCu/SnO_2_ electrodes for flexible and transparent thin film heaters

**DOI:** 10.1038/s41598-017-02711-2

**Published:** 2017-05-31

**Authors:** Doo-Hee Kim, Kyung-Su Cho, Han-Ki Kim

**Affiliations:** Kyung Hee University, Department of Advanced Materials Engineering for Information and Electronics, 1 Seocheon, Yongin, Gyeonggi-do 446-701 Republic of Korea

## Abstract

We investigated the characteristics of themally evaporated SnO_2_/Ag-Pd-Cu (APC)/SnO_2_ multilayer films for applications as damage-free, indium-free, flexible, and transparent electrodes for high performance flexible and transparent thin film heaters (TFHs). The top and bottom SnO_2_ layers and APC interlayer were prepared by a multi-source evaporation process, and the effect of the thickness of each layer on the resistivity, optical transmittance, and mechanical flexibility of the SnO_2_/APC/SnO_2_ electrodes was investigated in detail. Based on a figure of merit value, we obtained a SnO_2_/APC/SnO_2_ electrode with a low sheet resistance of 9.42 Ohm/square and a high optical transmittance of 91.14%. In addition, we examined the mechanical properties of the SnO_2_/APC/SnO_2_ electrode using various bending tests such as inner bending, outer bending, dynamic fatigue, and a twisting test. By comparing the crack shape of the SnO_2_/APC/SnO_2_ electrode bent beyond the critical bending radius (2~3 mm), we suggest a possible crack formation mechanism for the SnO_2_/APC/SnO_2_ electrodes. Furthermore, we evaluated the feasibility of the SnO_2_/APC/SnO_2_ electrodes for flexible and transparent TFHs. By correlating the sheet resistance of the SnO_2_/APC/SnO_2_ electrode and the performance of TFHs, we show the importance of transparent electrodes for high performance flexible and transparent TFHs.

## Introduction

The rapid advance of smart and functional window technology requires high performance flexible and transparent thin film heaters (TFHs), which can be attached on curved and transparent windows^1–3^. Because flexible and transparent TFHs can be employed in defogging/deicing windows and as heating sources for automobiles, displays, sensors, reaction cells, and microchips, much effort has been devoted to the development of high performance flexible and transparent TFHs^4, 5^. Among several components of TFHs, the transparent conductive layer plays an important role in the performance of TFHs because a TFH operates through the resistance heating of a transparent conductive layer. Therefore, a high-quality transparent conductive layer with low sheet resistance, high transparency, and good flexibility is necessary for high performance TFHs. In addition, thermal response time, steady heating temperature, operating voltage, temperature uniformity and cycling stability of TFHs are closely related to the quality of the transparent conductive layer. Until now, Sn-doped In_2_O_3_ (ITO) or F-doped SnO_2_ (FTO) films prepared by sputtering or chemical vapor deposition have been mainly employed as the transparent conductive layer in commercial transparent TFHs due to their low resistivity and high optical transmittance^6, 7^. Although both ITO and FTO films have low resistivity and high transmittance, the brittleness of the oxide-based transparent conductive layer is a critical problem for their use as flexible and transparent electrodes for flexible and transparent TFHs^8, 9^. In addition, the high cost of indium for the ITO film and the high process temperature for the FTO film is considered to be a limit for oxide-based transparent conductive electrodes. To substitute for conventional ITO and FTO films, several transparent conductive materials such as carbon-based electrodes (carbon nanotube, graphene, graphene oxides), conducting polymers, metal-based electrodes (Ag nanowires, Ag grids, Cu nanowires), and hybrid electrodes have been suggested as the transparent conductive layer for high performance TFHs^10–21^. In particular, oxide-metal-oxide (OMO) structures prepared by a continuous sputtering process have been reported as promising transparent conductive electrodes for organic light emitting diodes, organic solar cells, touch screen panels, and TFHs^22^. In the OMO structure, the highly conductive metal interlayer leads to a low resistivity, and the symmetric OMO structure can suppress reflection from the metal layer and results in high optical transmittance^23^. In our previous work, we also reported several indium-based OMO schemes such as ITO/Ag/ITO, ITO/Cu/ITO, InZnO/Ag/InZnO, InZnO/Au/InZnO, InZnSnO/Ag/InZnSnO, and InSiO/Ag/InSiO multilayer electrodes prepared by a sputtering process^24–28^. However, the high cost of indium and plasma damage on the flexible substrate or organic sublayer during the sputtering process remain as critical drawbacks. In addition, the plasma-based sputtered process can lead to degradation of the soft sublayers such as the organic layer or the flexible substrate due to severe bombardment of energetic particles in the high energy plasma^29^. Therefore, the development of an indium-free OMO electrode prepared by thermal evaporation is imperative to realize a low-cost and low-damage OMO electrode. Although several indium-free films including ZnSnO_3_/Ag/ZnSnO_3_, Ga-ZnO/Ag/Ga-ZnO, SnO_2_/Ag/SnO_2_ and FTO/Ag/FTO, have been suggested as transparent electrodes, they were also prepared by conventional DC or RF sputtering process^30–34^. In addition, while thermally evaporated indium-free WO_3_/Ag/WO_3_ or MoO_3_/Ag/MoO_3_ multilayer films have been reported as transparent cathodes for OLEDs, there have been no reports on thermally evaporated SnO_2_ with a nano-sized Ag-Pd-Cu (APC) interlayer for flexible and transparent TFHs. Because existence of the Pd and Cu elements in APC interlayer could improve the stability of Ag matrix and provide very flat surface of Ag layer, APC-based OMO structure is favorable to realize high quality OMO electrodes^35, 36^.

In this work, we reported the electrical, optical, and mechanical properties of a thermally evaporated SnO_2_/APC/SnO_2_ multilayer film on a PET substrate for use as a transparent conductive layer for high performance flexible and transparent TFHs. The electrical and optical properties of the SnO_2_/APC/SnO_2_ multilayer film were correlated with the thickness of top/bottom SnO_2_ layers and the APC interlayer, respectively. In addition, the mechanical flexibility of the SnO_2_/APC/SnO_2_ multilayer film was evaluated using a lab-made bending test system. Furthermore, the performance of flexible and transparent TFHs with the SnO_2_/APC/SnO_2_ electrodes was examined as a function of top/bottom SnO_2_ layer thickness to show the feasibility of thermally evaporated indium-free, plasma-damage free, flexible, and transparent SnO_2_/APC/SnO_2_ electrodes.

## Results

**Figure 1:**
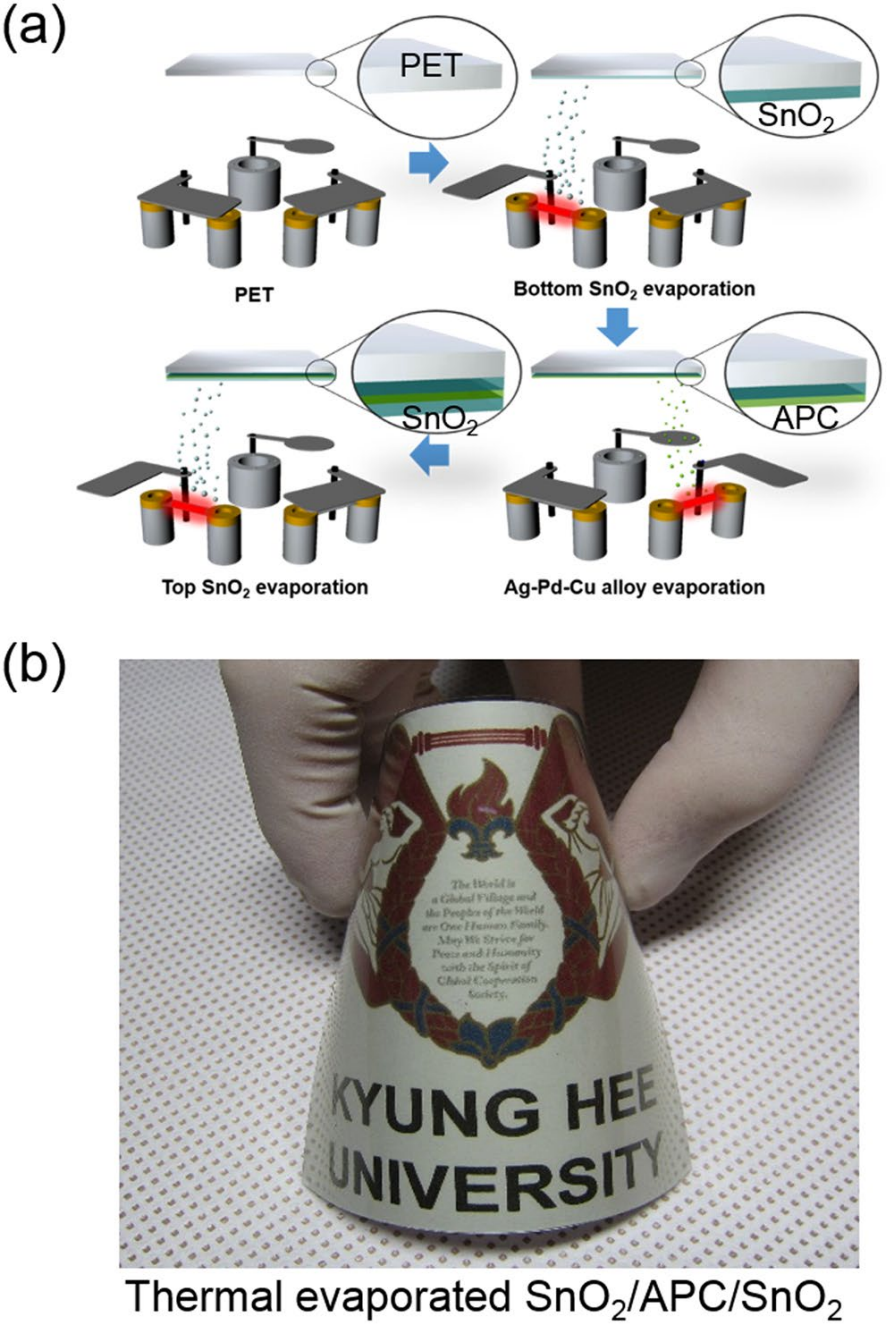
(**a**) Schematic process of the thermally evaporated SnO_2_/APC/SnO_2_ multilayer film on a PET substrate using a multi-boat evaporation system. (**b**) Photograph of the curved SnO_2_/APC/SnO_2_ multilayer film on a PET substrate demonstrating high optical transparency and good mechanical flexibility.

**Figure 2:**
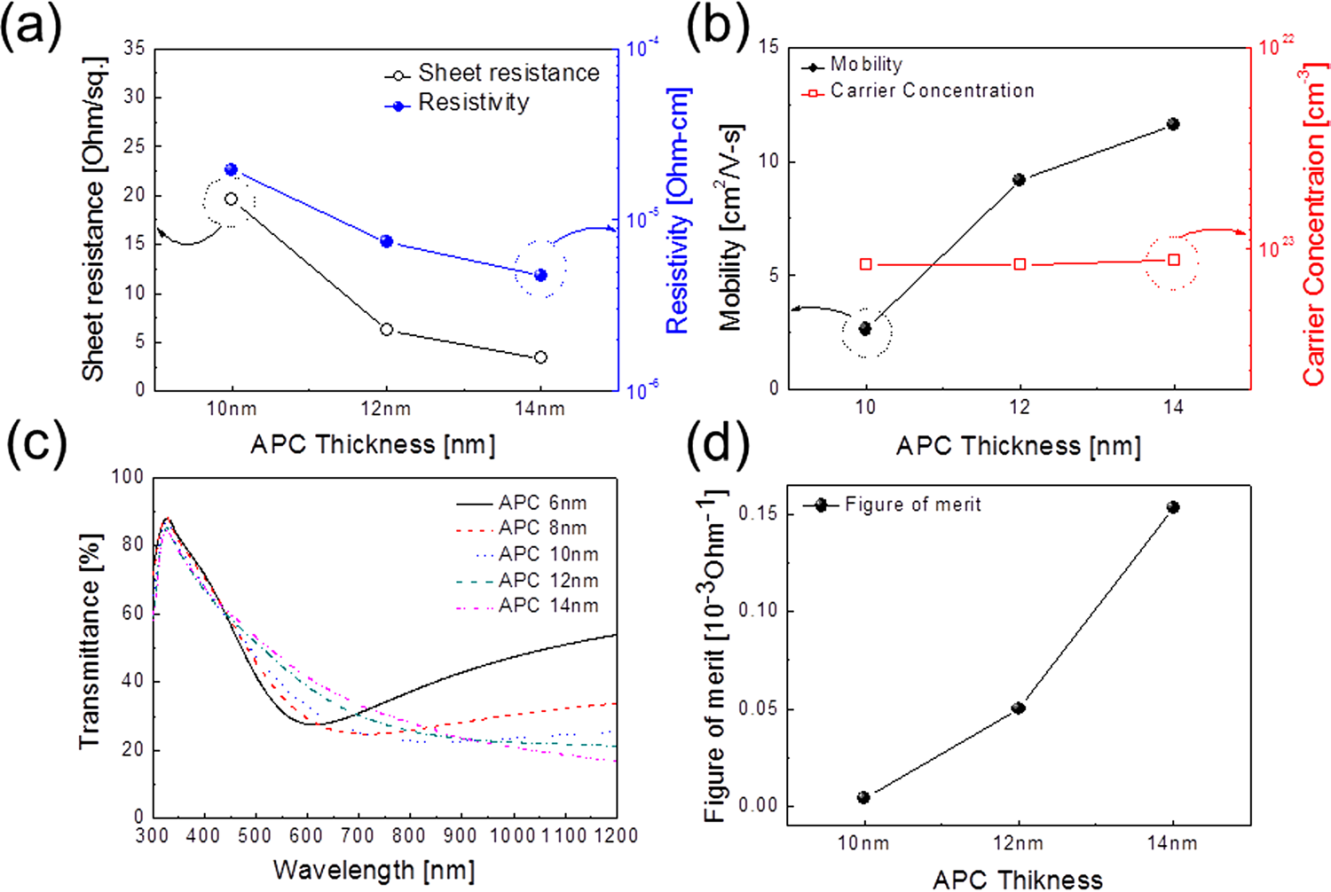
(**a**) Sheet resistance and resistivity and (**b**) mobility and carrier concentration of thermally evaporated APC layer on a PET substrate with increasing thickness from 6 to 14 nm. (**c**) Optical transmittance of the APC layer with increasing film thickness. (**d**) Figure of merit (FOM) values calculated from sheet resistance (R_sh_) and optical transmittance (T) of the APC films.

Figure 1a shows a schematic of the continuous thermal evaporation process used to fabricate a SnO_2_/APC/SnO_2_ multilayer film on a 125 μm-thick PET substrate without breaking vacuum. The SnO_2_/APC/SnO_2_ multilayer film consisted of three layers. First, the bottom SnO_2_ layer acts as an adhesion layer between the transparent multilayer film and the PET substrate. Second, the APC interlayer acts as the main conduction path during operation of TFHs and provides good flexibility in the SnO_2_/APC/SnO_2_ multilayer film. Finally, the top SnO_2_ layer acts as a symmetric oxide layer to realize the antireflection effect in the OMO structure. As illustrated in Fig. 1a, SnO_2_, APC, and SnO_2_ layers were continuously evaporated using multiple tungsten boats in the main evaporation chamber. The bottom and top SnO_2_ layers were coated by the left boat and the APC interlayer was coated by the right boat. Figure 1b shows a photograph of the thermally evaporated SnO_2_/APC/SnO_2_ (50/10/50 nm) multilayer film on a PET substrate. Due to a high optical transmittance above 90% and good flexibility, the symbol of Kyung Hee University behind the curved sample can be clearly seen. Before fabrication of the SnO_2_/APC/SnO_2_ full layer, we investigated the electrical and optical properties of the metallic APC interlayer as a function of its thickness. Figure 2a and b show Hall measurement results for the APC interlayer evaporated on a PET substrate with increasing thickness. Below an APC thickness of 10 nm, we could not obtain Hall measurement results for the APC interlayer due to agglomeration of the thermally evaporated APC films. It is well known that island growth is favorable in the initial growth region of a metal layer^37^. Unlike a metal layer on a glass substrate or oxide thin films, the APC layer easily agglomerated on the PET substrate even at thickness of 6 and 8 nm. Therefore, disconnected APC islands on the PET substrate cannot provide the effective conduction path for electrons. However, an APC thickness of 10 nm started to show a sheet resistance of 19.56 Ohm/square and resistivity of 1.9 × 10^−5^ Ohm·cm, indicating that the APC islands were connected. At an APC thickness of 14 nm, the film showed a sheet resistance of 3.37 Ohm/square and resistivity of 4.72 × 10^−6^ Ohm·cm, indicating metallic conduction. The decreased resistivity of the thin APC layer with increasing thickness could be mainly attributed to an increase in carrier mobility, as shown in Fig. 2b. The transition from an island-shaped APC film to a well-connected layered APC film led to decreases in surface and interface area, which are the main sources of scattering in the current flow^38^. Therefore, increased carrier mobility of the APC interlayer resulted in decreased sheet resistance and resistivity. Figure 2c shows the optical transmittance of the single APC layer as a function of APC thickness from 6 to 14 nm. Below a wavelength of 450 nm, the optical transmittance slightly decreased with increasing APC thickness, while at a wavelength between 450 and 700 nm, the optical transmittance increased with increasing APC thickness. By increasing the thickness of the APC layer from 6 to 14 nm, the optical transmittance at a wavelength of 550 nm increased from 31.4% to 46.91% due to decreasing severe light scattering of light in the APC layer. As expected from Hall measurement results, light scattering on the islands-shaped APC layer with a thickness below 8 nm led to low optical transmittance. Based on the sheet resistance (R_sh_) and transmittance (T) at a wavelength of 550 nm for the APC layer, the figure of merit (FOM = T^10^/R_sh_) values are calculated as a function of the thickness of the APC layer. As shown in Fig. 2d, the 14 nm-thick APC monolayer revealed the highest FOM value due to high optical transmittance and low sheet resistance. From the sheet resistance and optical transmittance of the APC layer, we determined that the thickness of the thermally evaporated APC layer should be greater than 10 nm to obtain high performance SnO_2_/APC/SnO_2_ multilayer electrodes. Table 1 summarizes the sheet resistance, optical transmittance, and FOM value of an APC single layer as a function of thickness.

**Table 1:** Sheet resistance and optical transmittance of a thermally evaporated APC monolayer grown on a PET substrate as well as the calculated figure of merit values.

Thickness	Sheet resistance [Ohm/sq.]	Transmittance_550__nm_ [%]	Figure of merit [10^−3^ Ohm^−1^]
6 nm	—	31.40	—
8 nm	—	35.73	—
10 nm	19.56	39.27	0.004
12 nm	6.221	44.62	0.050
14 nm	3.372	46.91	0.153

**Figure 3:**
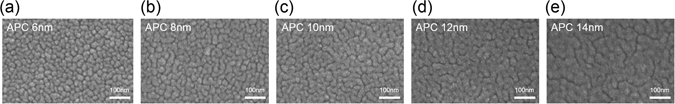
Surface FESEM images of thermally evaporated APC films on a PET substrate with a thickness of (**a**) 6 nm, (**b**) 8 nm, (**c**) 10 nm, (**d**) 12 nm, and (**e**) 14 nm, respectively.

To investigate the effect of APC thickness on the surface morphology, we employed field emission scanning electron microscopy (FESEM) analysis. Figure 3 shows surface FESEM images of the thermally evaporated APC layer as a function of thickness from 6 to 14 nm. By increasing the thickness of the APC layer from 6 to 14 nm, the surface of the APC layer changed from agglomerated islands to well-connected films. As expected from the Hall measurement results, the 6- and 8-nm-thick APC layers had disconnected APC islands, which prevent effective current flow, as shown in Fig. 3a and b. Therefore, we cannot obtain sheet resistance from the 6- or 8-nm-thick APC layers. The 10 nm-thick APC layer seen in Fig. 3c exhibited the surface image of a well-connected APC layer. Further increase in the thickness of the APC layer led to an increase in the connected APC area, which decreased the sheet resistance of the APC layer. Therefore, the decreased sheet resistance of the APC layer with increased thickness is well correlated with the connectivity of the APC islands.

**Figure 4:**
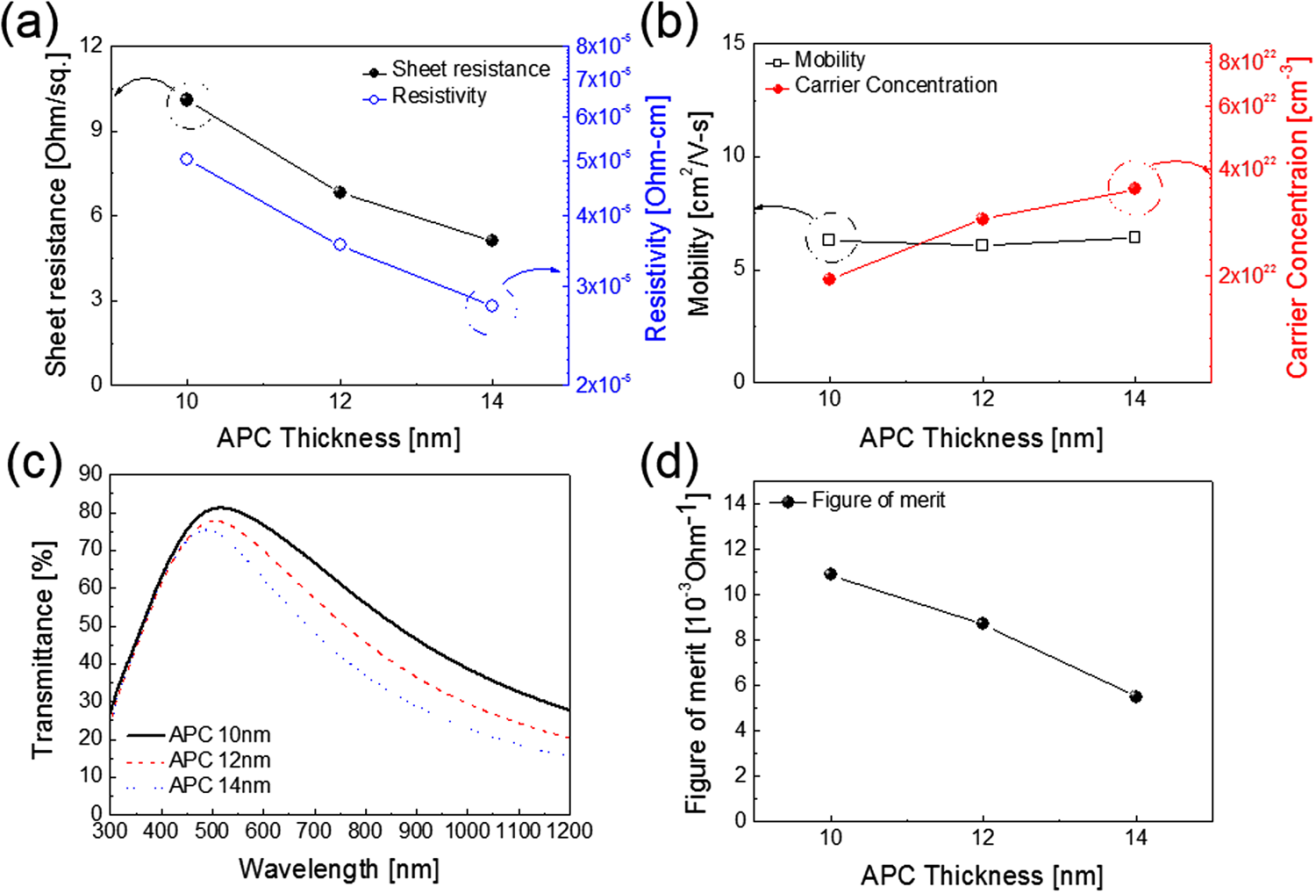
(**a**) Sheet resistance and resistivity and (**b**) mobility and carrier concentration of thermal evaporated SnO_2_/APC/SnO_2_ multilayer film on a PET substrate as a function of APC interlayer thickness at a constant thickness of SnO_2_ (20 nm). (**c**) Optical transmittance of the SnO_2_/APC/SnO_2_ multilayer film with different APC interlayers. (**d**) FOM values of the SnO_2_/APC/SnO_2_ multilayer film with different APC interlayer thicknesses.

Figure 4a and b show Hall measurement results of the thermally evaporated SnO_2_/APC/SnO_2_ multilayer film with increasing thickness of the APC interlayer (10, 12, 14 nm) with a fixed top and bottom SnO_2_ thickness of 20 nm. At the APC interlayer thickness of 10 nm, the SnO_2_/APC/SnO_2_ multilayer film showed a sheet resistance of 10.1 Ohm/square and a resistivity of 5.05 × 10^−5^ Ohm·cm even though the multilayer film was prepared at room temperature. An increase in APC interlayer thickness from 10 to 14 nm significantly decreased the sheet resistance (5.12 Ohm/square) and resistivity (2.77 × 10^−5^ Ohm·cm) of the SnO_2_/APC/SnO_2_ multilayer film. The sheet resistance and resistivity of the thermal evaporated SnO_2_/APC/SnO_2_ multilayer film are similar to those of sputtered SnO_2_/Ag/SnO_2_ multilayer film previously reported by Yu *et al*.^32^. The decreased resistivity of the SnO_2_/APC/SnO_2_ multilayer film could be explained by an increase in the carrier concentration, as shown in the Fig. 4b. With increasing APC interlayer thickness, the SnO_2_/APC/SnO_2_ multilayer film showed increased carrier concentration from 1.96 × 10^22^ to 3.51 × 10^22^ cm^−3^. As discussed by Alford *et al*., the APC interlayer can act as electron source for the top and bottom transparent conducting oxide layers^39^. However, the SnO_2_/APC/SnO_2_ multilayer film showed similar carrier mobility even though the APC interlayer thickness was different. Figure 4c shows the optical transmittance of the SnO_2_/APC/SnO_2_ multilayer film as a function of the APC interlayer thickness. Unlike the APC monolayer, the SnO_2_/APC/SnO_2_ multilayer film showed high optical transmittance due to the antireflection effect of the symmetric OMO structure^23, 40^. It was noteworthy that the SnO_2_/APC/SnO_2_ multilayer film with a 10 nm-thick APC interlayer showed the highest optical transmittance of 80.18% at a wavelength of 550 nm, unlike the optical transmittance of the APC monolayer. Because the SnO_2_/APC/SnO_2_ structure can suppress reflection from the APC interlayer, the SnO_2_/APC/SnO_2_ multilayer film showed higher optical transmittance than the APC single layer with the same thickness. However, with increasing thickness of the APC interlayer, the optical transmittance of the SnO_2_/APC/SnO_2_ multilayer film decreased. In the SnO_2_/APC/SnO_2_ multilayer, interference effect resulted in the wave reflected from the top surface of SnO_2_/APC/SnO_2_ film to be out of phase with the wave reflected from APC layer and PET substrate. Therefore, control of the optimal top/bottom SnO_2_ and APC thickness is very important to obtain high optical transmittance from the multilayer. In addition, the increase in thickness of the APC interlayer in the SnO_2_/APC/SnO_2_ multilayer film led to a bluish color of the sample. Based on sheet resistance and optical transmittance at a wavelength of 550 nm for the SnO_2_/APC/SnO_2_ multilayer film, the FOM values were calculated as a function of the APC interlayer. As shown in Fig. 4d, the 10 nm-thick SnO_2_/APC/SnO_2_ multilayer film revealed the highest FOM value of 3.57 × 10^−3^ Ohm^−1^, due to the highest optical transmittance of 80.18% despite the slightly higher sheet resistance.

**Figure 5:**
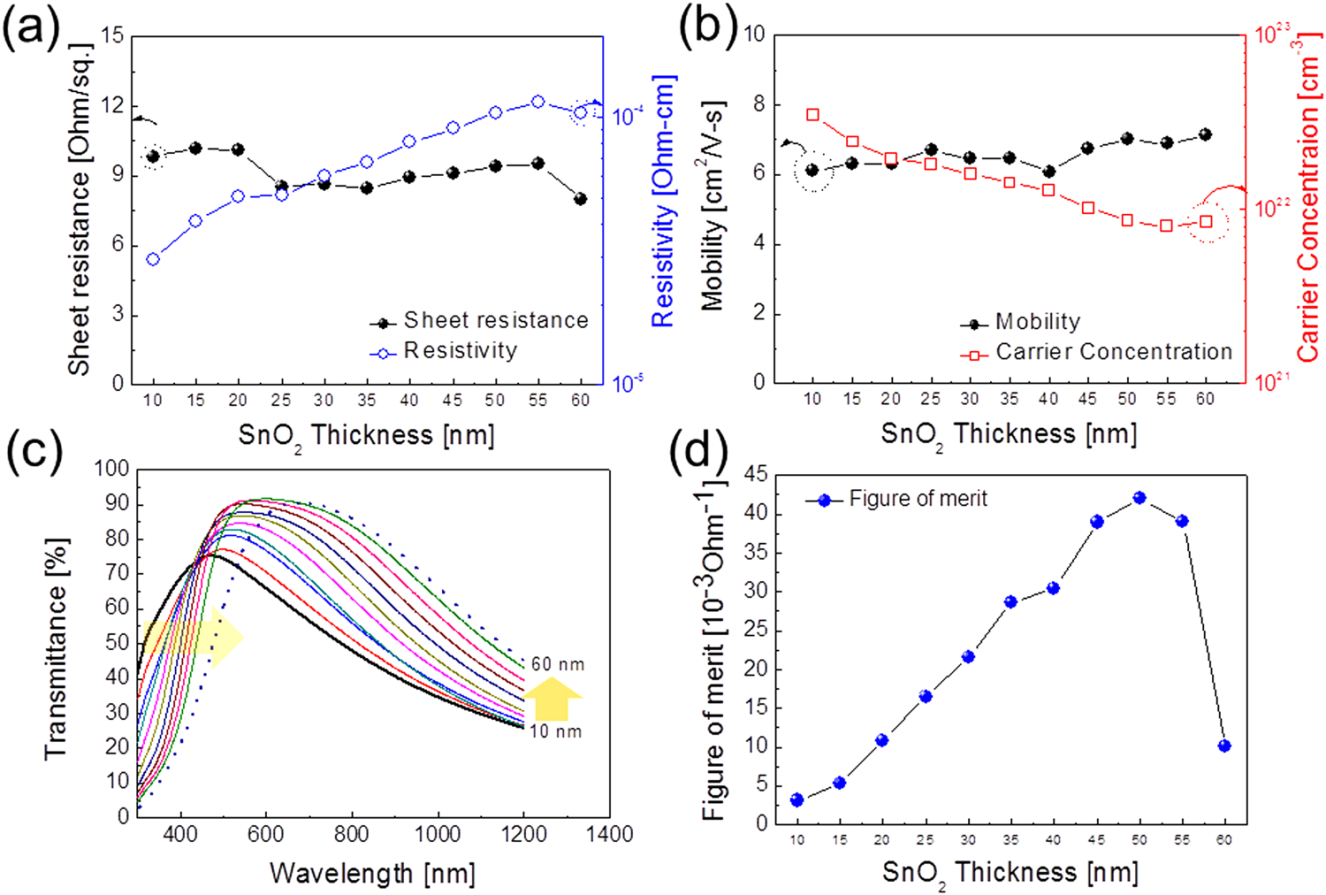
(**a**) Sheet resistance and resistivity and (**b**) mobility and carrier concentration of the thermally evaporated SnO_2_/APC/SnO_2_ multilayer film on a PET substrate as a function of the bottom and top SnO_2_ layer thicknesses at a constant thickness (10 nm) for the APC inter layer. (**c**) Optical transmittance and (**d**) FOM values of the SnO_2_/APC/SnO_2_ multilayer film as a function of the bottom and top SnO_2_ layer thicknesses.

Figure 5a and b show the Hall measurement results of the SnO_2_/APC/SnO_2_ multilayer film with increasing thickness of the top and bottom SnO_2_ layers at a fixed APC thickness of 10 nm. It was noteworthy that all SnO_2_/APC/SnO_2_ multilayer films showed a similar sheet resistance (8.00–10.19 Ohm/square) at a constant APC interlayer thickness regardless of the top and bottom SnO_2_ layers thicknesses because the main conduction path in SnO_2_/APC/SnO_2_ is the metallic APC layer. However, the resistivity of the SnO_2_/APC/SnO_2_ multilayer film increased linearly with increasing top and bottom SnO_2_ layer thicknesses. The existence of thicker top and bottom SnO_2_ layers with high resistivity led to an increase in resistivity of the SnO_2_/APC/SnO_2_ multilayer film, as shown in Fig. 5a. The increased resistivity could be attributed to the decreased carrier concentration, as shown in Fig. 5b. Compared to a fairly constant carrier mobility, the carrier concentration of the SnO_2_/APC/SnO_2_ multilayer film linearly decreased because the overall thickness was increased. Due to an increase in the top and bottom SnO_2_ layer volume at a constant APC thickness, the carrier concentration injected from the metal APC interlayer decreased, and this increased the resistivity of the SnO_2_/APC/SnO_2_ multilayer film. Yu *et al*. also noted a similar thickness dependence for the top and bottom FTO layers on the resistivity of FTO/Ag/FTO multilayer electrodes^34^. Figure 5c shows the optical transmittance of the SnO_2_/APC/SnO_2_ multilayer film with increasing top and bottom SnO_2_ layer thicknesses. The increase in the thicknesses of the top and bottom SnO_2_ layers from 10 to 50 nm led to an improvement in optical transmittance. It was clearly shown that the SnO_2_/APC/SnO_2_ multilayer film with 50 nm-thick top and bottom SnO_2_ layers has the highest optical transmittance of 91.14% at a wavelength of 550 nm. However, a further increase in the thickness of the top and bottom SnO_2_ layers results in a decrease in the optical transmittance. In addition, all multilayer films showed a sharp absorption onset in the near-UV region. With increasing top and bottom SnO_2_ layer thickness, the absorption edge of the multilayer film shifted to longer wavelengths, as indicated by the arrow in Fig. 5c. As discussed by Supasai *et al*., the broadening of the absorption edge for transparent films is closely related to imperfection and disorder in the films^41^. Although the SnO_2_/APC/SnO_2_ multilayer showed high optical transmittance in a visible wavelength region (400–800 nm), the optical transmittance of the SnO_2_/APC/SnO_2_ multilayer in near IR region is fairly low due to existence of metallic APC interlayer. Low near IR transmittance of the SnO_2_/APC/SnO_2_ multilayer can be used as an insulating widow film preventing the heat flow through window. Based on the sheet resistance and optical transmittance at a wavelength of 550 nm for the SnO_2_/APC/SnO_2_ multilayer film, FOM values were also calculated as a function of thickness of the top and bottom SnO_2_ layer, as shown in Fig. 5d. The SnO_2_/APC/SnO_2_ multilayer film with a 50 nm-thick SnO_2_ layer showed the highest FOM value of 41.98 × 10^−3^ Ohm^−1^ due to a low sheet resistance of 9.42 Ohm/square and a high optical transmittance of 91.14% at a wavelength of 550 nm. Table 2 summarizes the sheet resistance, optical transmittance, and FOM value of the SnO_2_/APC/SnO_2_ multilayer film as a function of the top and bottom SnO_2_ layer thickness.

**Table 2:** Sheet resistance and optical transmittance of thermally evaporated SnO_2_/APC/SnO_2_ multilayer film grown on a PET substrate as well as the calculated figure of merit as a function of SnO_2_ layer thickness at a fixed APC thickness of 10 nm.

SnO_2_ Thickness	Sheet resistance [Ohm/sq.]	Transmittance_550__nm_ [%]	Figure of merit [10^−3^ Ohm^−1^]
10 nm	9.83	70.59	3.12
15 nm	10.19	74.75	5.34
20 nm	10.10	80.18	10.87
25 nm	8.54	82.20	16.49
30 nm	8.65	84.55	21.58
35 nm	8.48	86.78	28.56
40 nm	8.94	87.79	30.42
45 nm	9.08	90.13	38.96
50 nm	9.42	91.14	41.98
55 nm	9.51	90.56	39.01
60 nm	8.00	77.77	10.12

**Figure 6:**
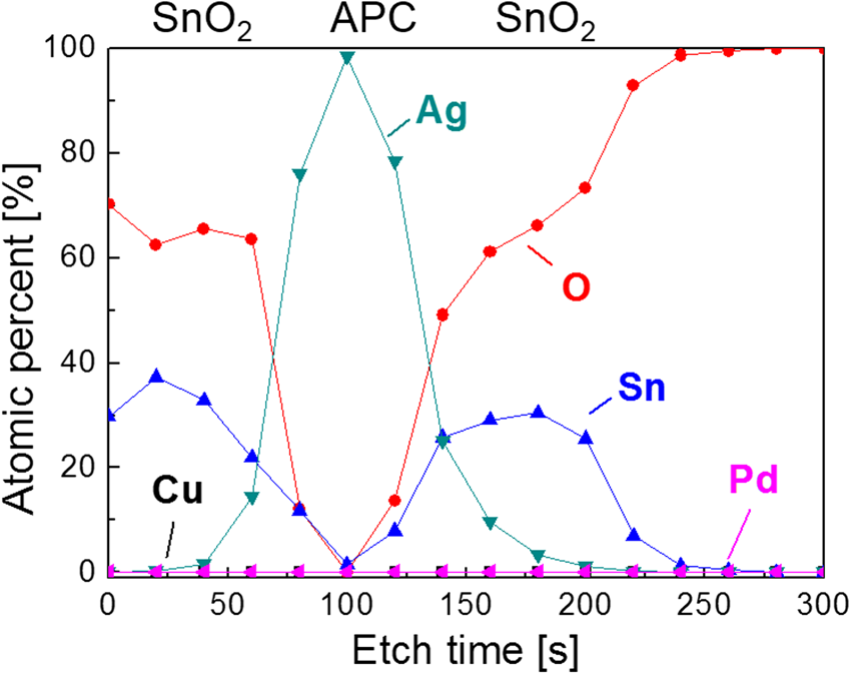
XPS depth profile of the thermally evaporated SnO_2_/APC/SnO_2_ multilayer film on a PET substrate.

Figure 6 shows the X-ray photoelectron spectroscopy (XPS) depth profile of the thermally evaporated SnO_2_/APC/SnO_2_ multilayer film with a thickness of 20 nm/10 nm/20 nm. The XPS depth profile showed a symmetric oxide-metal-oxide structure due to identical SnO_2_ evaporating processes. In addition, a well-defined interface between SnO_2_ and the APC interlayer without carbon contamination indicates that there are no interface reactions between the APC interlayer and the top/bottom SnO_2_ layers. All evaporation processes were carried out at room temperature with an intentional substrate cooling system, so there was no interfacial reaction between SnO_2_ and the metallic APC layer. Due to well-defined OMO structure without a diffused interface, we expected an effective antireflection effect in the SnO_2_/APC/SnO_2_ multilayer film.

**Figure 7:**
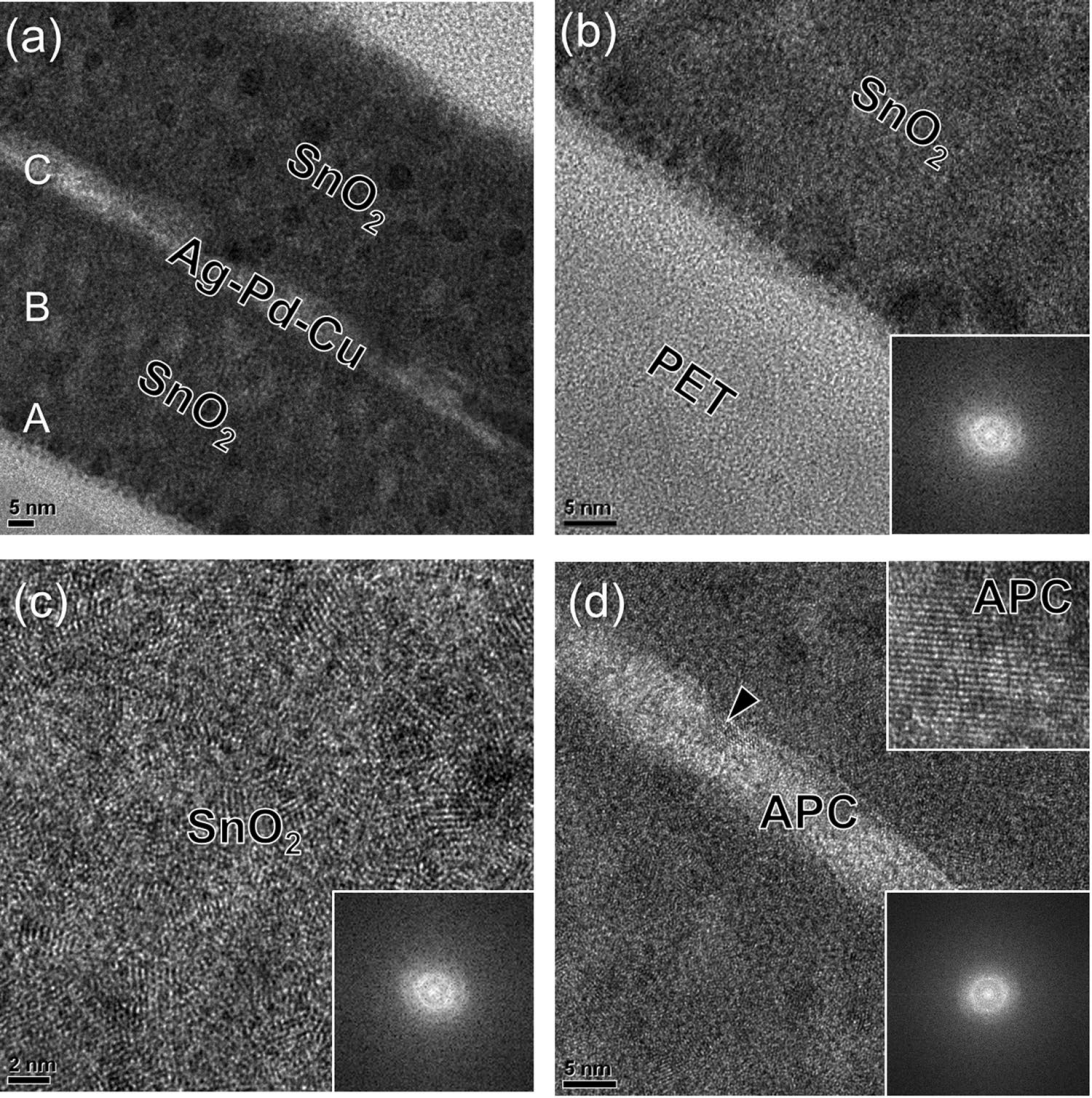
(**a**) Cross-sectional TEM image of the thermally evaporated SnO_2_/APC/SnO_2_ multilayer film on a PET substrate. Enlarged TEM images obtained from A, B, and C in the cross-sectional image with an inset of FFT patterns; (**b**) PET/SnO_2_ interface region, (**c**) bottom SnO_2_ region, and (**c**) SnO_2_/APC/SnO_2_ interface region, respectively.

To investigate the microstructure of the thermally evaporated SnO_2_/APC/SnO_2_ multilayer film, transmittance electron microscopy (TEM) was employed. Figure 7a shows a cross-sectional TEM image of the SnO_2_/APC/SnO_2_ (50/10/50 nm) multilayer film evaporated on a PET substrate. As expected from the XPS depth profile, the symmetric OMO structure was observed in the cross-sectional TEM image. In addition, the APC interlayer connected the top SnO_2_ layer and bottom SnO_2_ layer without any disconnection due to a very flat surface morphology. As we reported in our previous work, the APC layer had a flatter surface morphology than a pure Ag layer due to the existence of Pd element^42^. Therefore, in the thermally evaporated SnO_2_/APC/SnO_2_ multilayer, we expected a very flat APC interlayer, unlike the typical pure Ag interlayer in an OMO structure. The cross-sectional TEM image clearly showed a well-defined interface between APC and the SnO_2_ layer with no evidence of interfacial reactions. However, there are some dark blobs in the top and bottom SnO_2_ layers, indicating embedded SnO_2_ nanocrystallines. The enlarged cross-sectional image obtained from the interface between the PET and bottom SnO_2_ layer in Fig. 7b clearly demonstrated that the as-deposited bottom SnO_2_ layer had an amorphous structure as expected from the diffuse fast Fourier transform (FFT) pattern in the inset of Fig. 7b. The enlarged TEM image (Fig. 7c) obtained from the middle of the bottom SnO_2_ layer also showed an amorphous structure with inset of the diffuse FFT pattern. During the thermal evaporation process, the PET substrate temperature was kept constant at 25 °C by an intentional substrate cooling system, and the bottom SnO_2_ film showed a completely amorphous structure. However, the APC interlayer existed in a crystalline form as shown in the enlarged TEM image in Fig. 7d and the FFT pattern with bright dots. Even though the APC layer was evaporated at room temperature, it had a crystalline structure, as indicated by the arrow and inset image.

**Figure 8:**
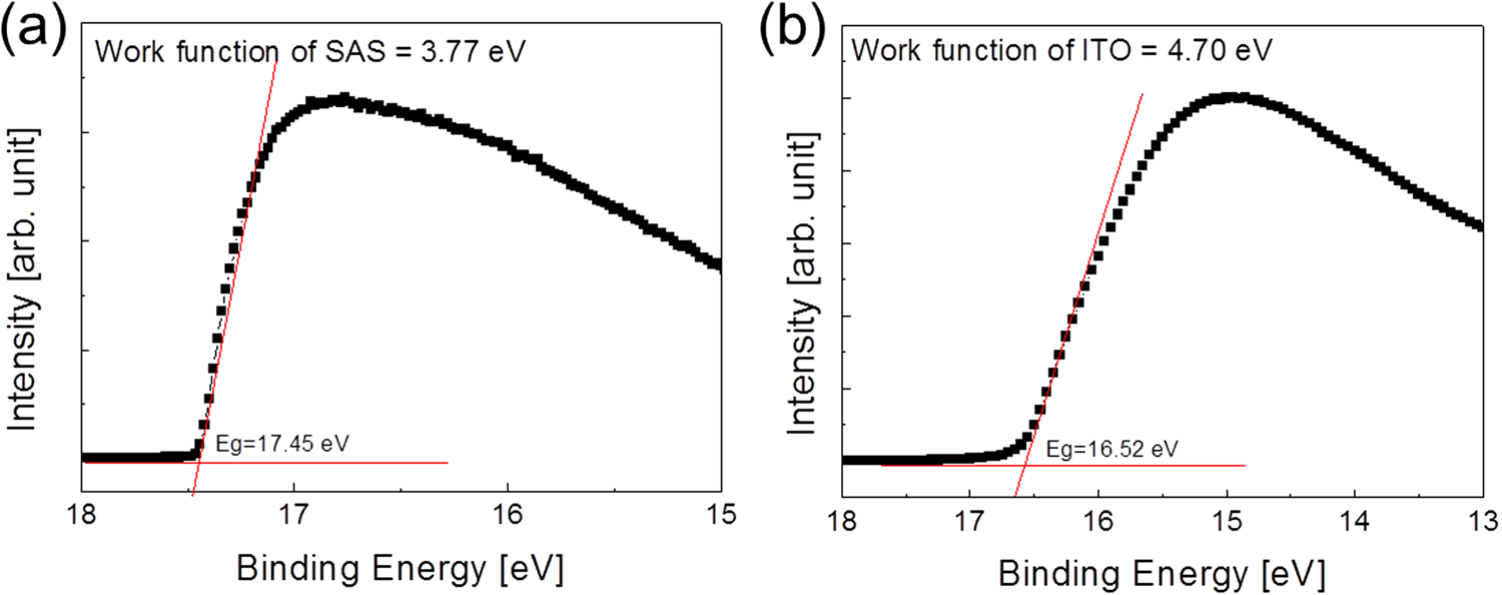
Work function of (**a**) SnO_2_/APC/SnO_2_ multilayer and (**b**) ITO electrode obtained from UPS analysis.

The work function of the SnO_2_/APC/SnO_2_ (50/10/50) multilayer was measured by ultraviolet photoelectron spectroscopy (UPS) as shown in Fig. 8. The work function (*Φ*) associated with the Fermi level (*E*_*F*_) is determined by the relation, *Φ* = photon energy (21.22 eV) − binding energy of the secondary cutoff in the UPS spectra (Fig. 8). The work function of the SnO_2_/APC/SnO_2_ multilayer (3.77 eV) was much lower than that of ITO film (4.7 eV). Therefore, the low work function of the SnO_2_/APC/SnO_2_ multilayer indicates the possibility of the SnO_2_/APC/SnO_2_ layer as an electron injection electrode (cathode) for transparent organic light emitting diodes.

**Figure 9:**
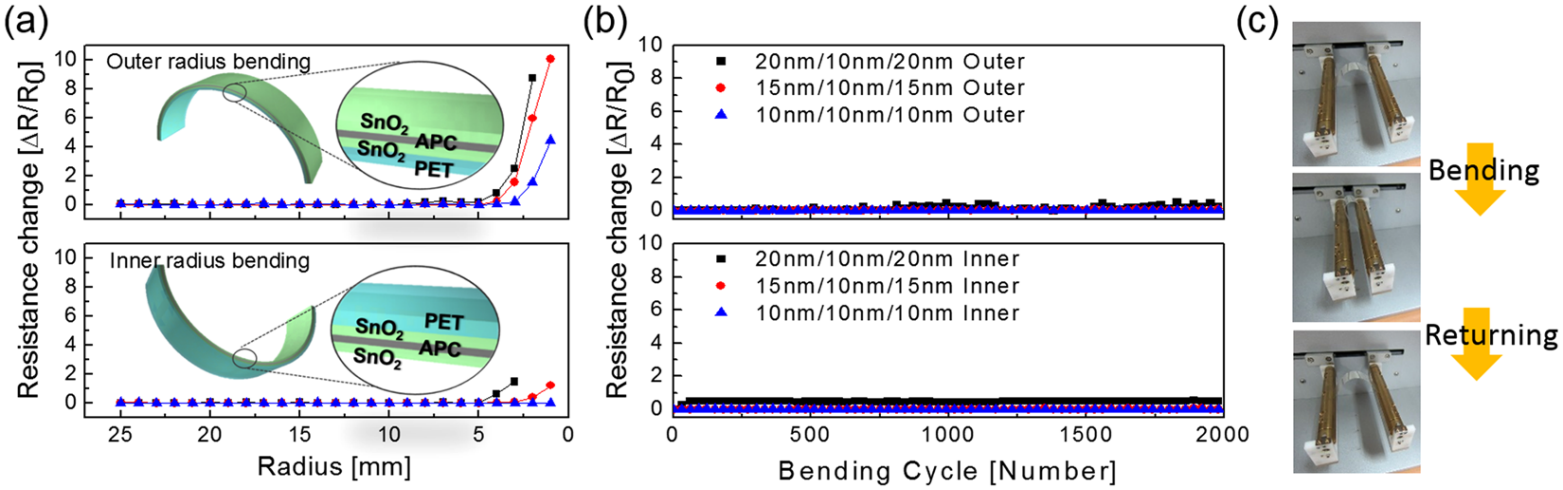
(**a**) Inner and outer bending and (**b**) dynamic fatigue tests of the thermally evaporated SnO_2_/APC/SnO_2_ multilayer film as a function of the top and bottom SnO_2_ layer thickness. (**c**) Pictures show the bending steps during the dynamic fatigue test.

The mechanical flexibility of the thermally evaporated SnO_2_/APC/SnO_2_ multilayer film was investigated by a lab-designed inner and outer bending test system. Figure 9a shows the results of the outer/inner bending tests for the SnO_2_/APC/SnO_2_ multilayer electrodes with decreasing outer/inner bending radii. At a constant APC thickness of 10 nm, we varied the top and bottom SnO_2_ layer thickness from 10 to 20 nm in the SnO_2_/APC/SnO_2_ multilayer. Although the SnO_2_/APC/SnO_2_ multilayer film with a 50 nm-thick SnO_2_ layer showed the highest FOM value, considering the mechanical flexibility, the thin bottom and top layers are more favorable to the mechanical flexibility of the SnO_2_/APC/SnO_2_ multilayer film. Therefore, we selected the SnO_2_/APC/SnO_2_ multilayer film samples with a SnO_2_ thickness below 20 nm. During substrate inner/outer bending, we measured the change of resistance (ΔR = R − R_0_) with decreasing bending radius, where R_0_ is the initial measured resistance of the sample, and R is the resistance measured under SnO_2_/APC/SnO_2_/PET bending. The outer bending test result (upper panel) shown in Fig. 9a illustrates that the SnO_2_/APC/SnO_2_ multilayer film with a 20 nm-thick SnO_2_ layer had a constant resistance until the bending radius reached 4 mm. Further decrease of the top and bottom SnO_2_ layer thickness led to a decrease of the critical bending radius for the SnO_2_/APC/SnO_2_ multilayer film to 3 and 2 mm, respectively. The following equation can be used to calculate the peak strain for a curved SnO_2_/APC/SnO_2_ multilayer film with decreasing bending radius^43^:

1 $${\rm{Strain}}=\frac{{{\rm{d}}}_{{\rm{f}}}+{{\rm{d}}}_{{\rm{PET}}}}{2{\rm{R}}}\times 100$$

Here, *d*_f_ and *d*_PET_ are the thicknesses of the SnO_2_/APC/SnO_2_ multilayer film and the PET substrate, respectively. Bending the SnO_2_/APC/SnO_2_ multilayer film (20 nm/10 nm/20 nm) on a 125 μm-thick PET substrate to a bending radius of 4 mm resulted in a peak strain of 1.56%. In addition, the SnO_2_/APC/SnO_2_ sample with 15 and 10 nm-thick SnO_2_ layers resulted in a peak strain of 2.08% and 3.13%, respectively. Beyond the critical bending radius, the resistance of the SnO_2_/APC/SnO_2_ multilayer film rapidly increased due to crack formation and propagation. In the inner bending tests, the measured resistance of the SnO_2_/APC/SnO_2_ multilayer film with 20 and 15 nm-thick SnO_2_ layers was constant until the sample was bent to an inner bending radius of 3 and 2 mm, respectively. However, the SnO_2_/APC/SnO_2_ multilayer film with a 10 nm-thick SnO_2_ layer showed no change of resistance even though cracks were formed on the sample. Under compressive stress as illustrated in the inset picture of Fig. 9a, the flexible SnO_2_/APC/SnO_2_ multilayer film showed constant resistance due to overlapping or physical contact of the cracked or delaminated layers. Figure 9b shows the dynamic outer and inner bending fatigue test results for the SnO_2_/APC/SnO_2_ multilayer film with increasing bending cycles at a fixed bending radius of 5 mm and repeating rate of 1 Hz. Figure 9c shows images of the dynamic outer/inner bending test step of the SnO_2_/APC/SnO_2_ sample. Both dynamic outer and inner bending fatigue tests showed no change in resistance (ΔR) during 2,000 bending cycles, demonstrating the good flexibility of the SnO_2_/APC/SnO_2_ multilayer film. Even when the samples were flexed near the critical bending radius (~5 mm), they showed constant resistance, indicating the feasibility of the SnO_2_/APC/SnO_2_ multilayer film for highly flexible electrodes in TFHs. The superior flexibility of the thermally evaporated SnO_2_/APC/SnO_2_ multilayer film is closely related to the high strain failure of the metallic APC layer and the thin amorphous SnO_2_ layer. Based on the electrical and optical analysis results of the thermal evaporated SnO_2_/APC/SnO_2_ multialyer as well as mechanical tests, we can find the oxide/metal/oxide configuration is beneficial to obtain highly flexible and transparent electrodes with a very low sheet resistance. Insertion of metallic APC layer into the SnO_2_ layer is effective way to reduce the resistivity and improve the mechanical flexibility of the evaporated SnO_2_ films. In addition, dielectric/metal/dielectric configuration could realize high optical transmittance comparable to conventional ITO or FTO electrode.

**Figure 10:**
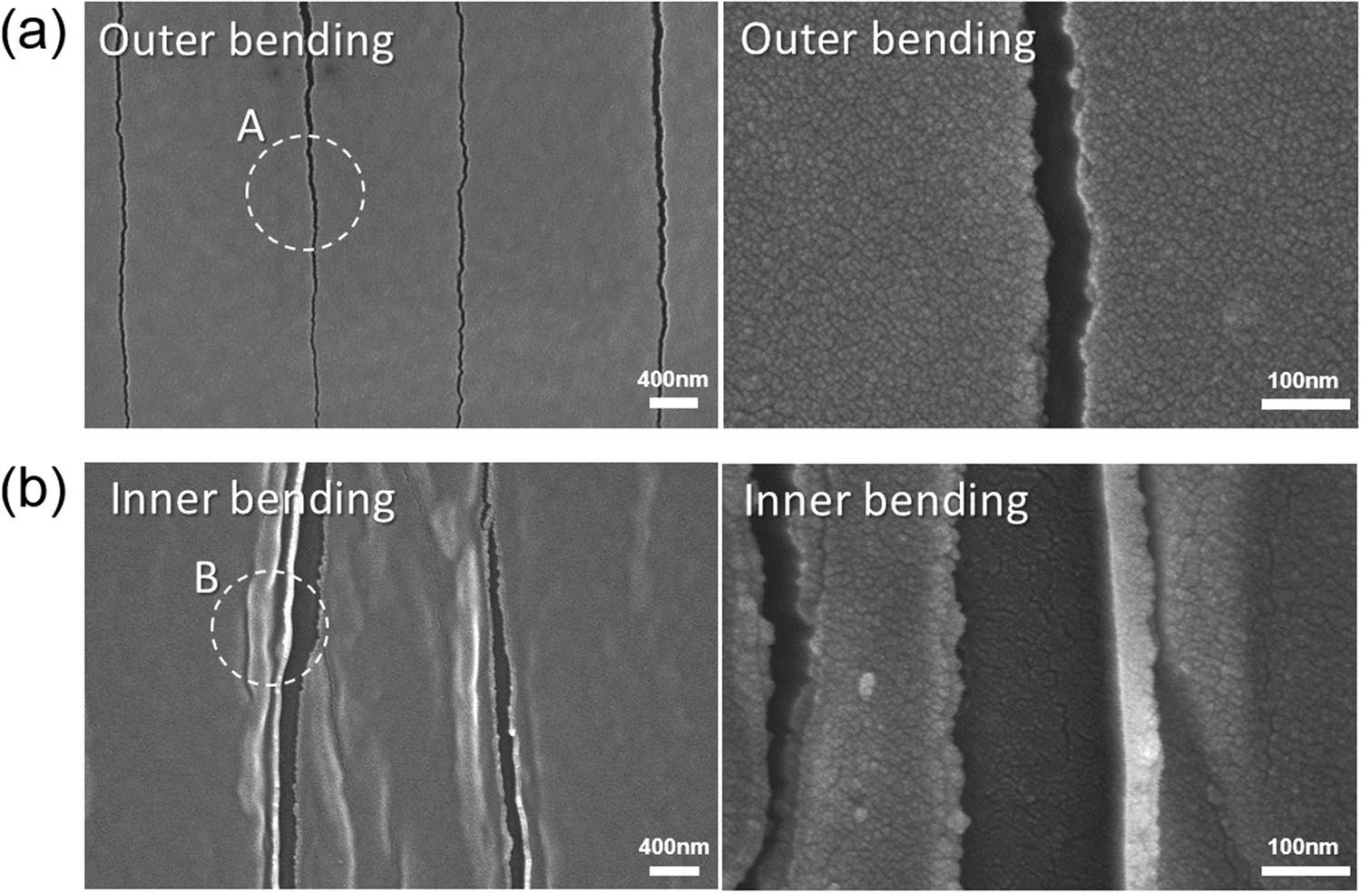
Surface FESEM image and enlarged image of the SnO_2_/APC/SnO_2_ multilayer after the (**a**) outer bending (2 mm) and (**b**) inner bending (1 mm) tests beyond the critical bending radius.

To study the surface morphology of the SnO_2_/APC/SnO_2_ multilayer film after crack formation beyond the critical bending radius, we employed FESEM analysis. Figure 10a shows the surface morphology of the SnO_2_/APC/SnO_2_ (20 nm/10 nm/20 nm) multilayer sample that was subjected to an outer bending test with a bending radius 2 mm. There are several cracks on the SnO_2_/APC/SnO_2_ multilayer film perpendicular to the bending direction. Because the outer bending test applied severe tensile stress to the sample, the crack was propagated in the perpendicular direction. The enlarged FESEM image clearly shows that the cracks on the sample separated the SnO_2_/APC/SnO_2_ multilayer film and increased the measured resistance change during the outer bending test. Figure 10b shows the surface morphology of the SnO_2_/APC/SnO_2_ (20 nm/10 nm/20 nm) multilayer sample after an inner bending test with a bending radius of 1 mm. Even though the sample shows a small resistance change in Fig. 9a, there are several cracks in the SnO_2_/APC/SnO_2_ multilayer film. Unlike the outer bending test, the cracks of inner bending test have a ‘hill’ near the edge due to overlapping or physical contact of the cracked region during the inner bending test. During the inner bending test with a very small inner bending radius of 1 mm, the SnO_2_/APC/SnO_2_ multilayer film experienced compressive stress. Several cracks were formed by compressive stress and propagated in the direction perpendicular to the inner bending direction, as shown in Fig. 10b. However, the physically overlapped region could conduct the current during the inner bending test, so the inner bent sample showed a constant resistance change even though the sample was severely cracked.

**Figure 11:**
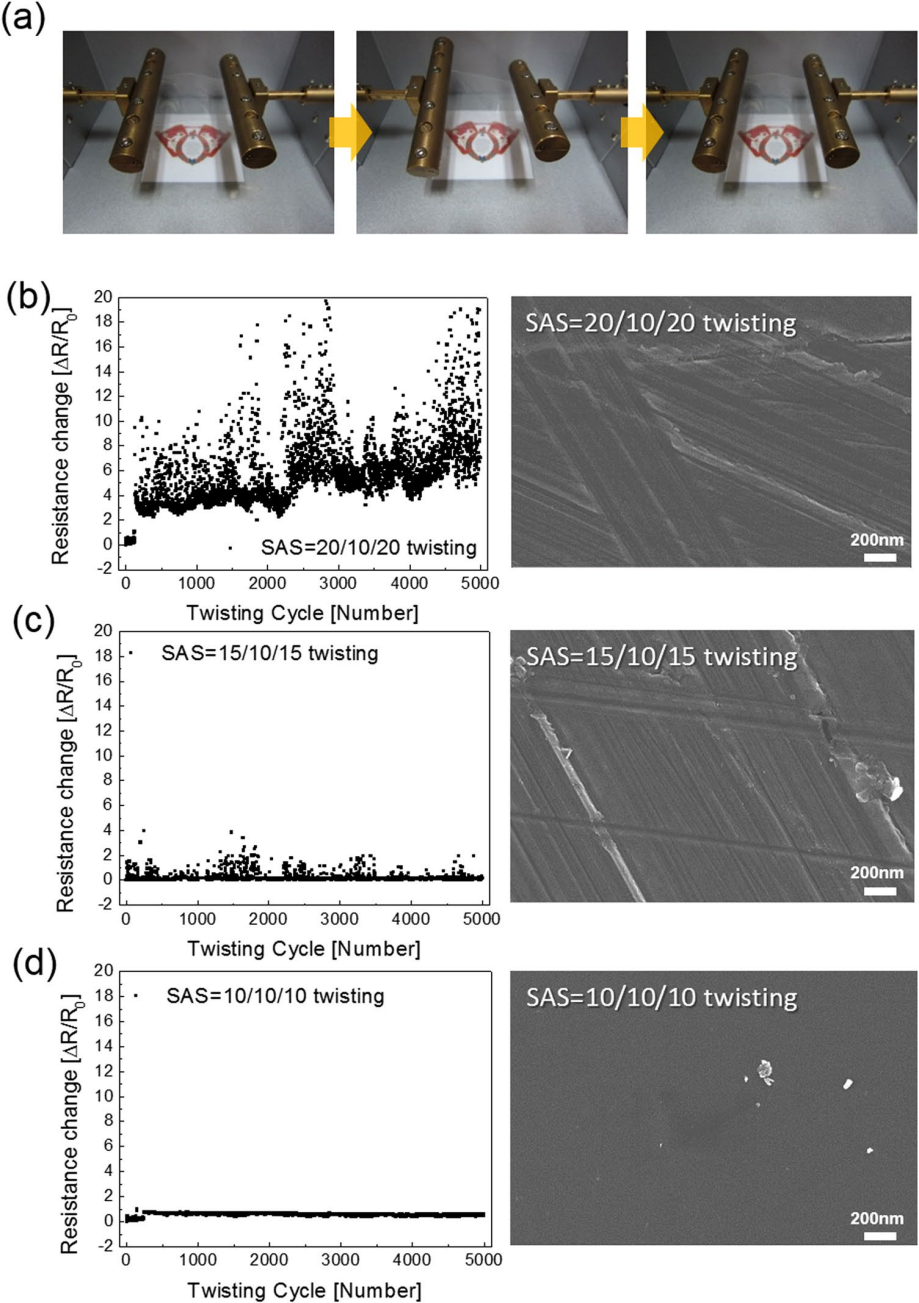
(**a**) Images of the twisting steps using a lab-designed twisting test system. The resistance change during the twisting test and surface FESEM images of the thermally evaporated SnO_2_/APC/SnO_2_ multilayer with SnO_2_ thickness of (**b**) 20 nm, (**c**) 15 nm, and (**d**) 10 nm respectively.

As another mechanical flexibility test, we employed a twisting test. Figure 11a shows images of the lab-designed twisting test steps. By twisting the sample, we investigated the mechanical stability of the transparent electrode with twisting. Figure 11b–d exhibit the resistance change of the SnO_2_/APC/SnO_2_ multilayer samples with increasing twisting cycles at a fixed twisting angle 15° and surface FESEM images after the twisting test. In the case of the SnO_2_/APC/SnO_2_ multilayer film with a SnO_2_ thickness of 20 nm, the resistance change (ΔR/R_0_) was severely increased for the twisting test performed 5,000 times. The resistance change indicated the formation of a crack during the twisting test. The surface FESEM image clearly shows the cross-shaped cracks on the SnO_2_/APC/SnO_2_ multilayer film, which were formed when the sample was severely twisted. In the case of the SnO_2_/APC/SnO_2_ multilayer film with a 15 nm-thick SnO_2_ layer, Fig. 11c shows a similar resistance change and surface FESEM image. The formation of cross-shaped cracks led to an increase of resistance change in the SnO_2_/APC/SnO_2_ multilayer film. Throughout the 5,000 twisting cycles, the ΔR/R_0_ value of the SnO_2_/APC/SnO_2_ multilayer film changed from 0 to 4. However, the SnO_2_/APC/SnO_2_ sample with 10 nm-thick SnO_2_ layer showed a constant resistance change even though it was twisted 5000 times. In the surface FESEM image, there were no cross-shaped cracks on the surface of the top SnO_2_ layer. As the thickness of top and bottom SnO_2_ layers became thinner, the ΔR/R_0_ value for the SnO_2_/APC/SnO_2_ multilayer film decreased.

**Figure 12:**
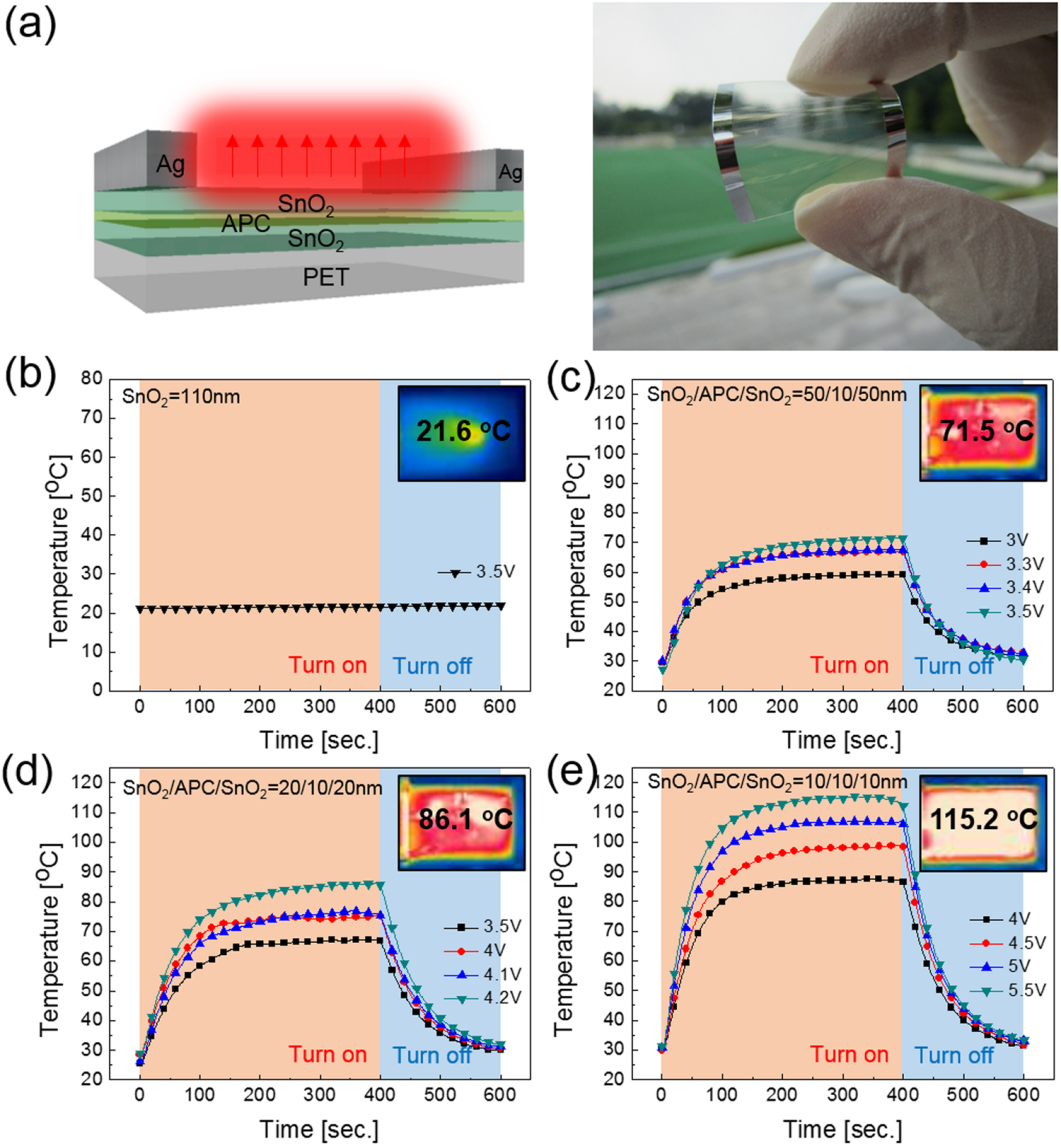
(**a**) Schematic structure and picture of flexible and transparent thin film heaters fabricated with thermally evaporated SnO_2_/APC/SnO_2_ multilayer electrodes. Temperature profile of flexible and transparent TFHs fabricated on the SnO_2_/APC/SnO_2_ multilayer film as a function of the SnO_2_ thickness; (**b**) Single SnO_2_ layer, (**c**) 50 nm-thick SnO_2_, (**d**) 20 nm thick-SnO_2_ and (**e**) 10 nm-thick SnO_2_ respectively. The inset shows the IR images at the saturation temperature.

To investigate the feasibility of the thermal evaporated SnO_2_/APC/SnO_2_ multilayer film as a flexible and transparent electrode for TFHs, SnO_2_/APC/SnO_2_ multilayer film-based TFHs were fabricated with a size of 50 × 50 mm using a two-terminal Ag contact configuration. Figure 12a shows the schematic structure and image of TFHs fabricated on thermally evaporated SnO_2_/APC/SnO_2_ multilayer electrodes. DC voltage was applied to the TFHs by a power supply through Ag metal contact electrodes at the film edge, and the temperature profile of the TFHs was measured by a thermocouple placed on the surface of the TFHs. In addition, an infrared (IR) thermal image was obtained using an IR thermometer. Figure 12b–e show the temperature profiles of the SnO_2_/APC/SnO_2_ multilayer based-TFHs, plotted with respect to input voltage as a function of a SnO_2_ layer thickness 50, 20, and 10 nm. As a reference sample, we prepared a 110 nm-thick SnO_2_ electrode fabricated through thermal evaporation. In general, as the input voltage was increased, the temperature of the TFHs increased, as shown in all of the temperature profiles except the reference SnO_2_-based TFH. Due to the high resistivity of the thermally evaporated SnO_2_ single layer, the reference SnO_2_-based TFHs did not show the same temperature profile as shown in Fig. 12b. However, insertion of a metallic APC layer in the SnO_2_ layer led to operation of TFHs due to significantly reduced resistivity. The TFH fabricated on SnO_2_/APC/SnO_2_ (50/10/50 nm) reached 71.5 °C when the DC voltage supplied was 3.5 V. However, the TFH burned out when a voltage above 3.5 V was supplied to the sample. In the case of the TFH fabricated on SnO_2_/APC/SnO_2_ (20/10/20 nm), 4.2 V of input DC voltage was necessary in order to reach at saturation temperature of 86.1 °C. Although the TFH with the SnO_2_/APC/SnO_2_ (20/10/20 nm) electrode showed a higher temperature than the TFH with SnO_2_/APC/SnO_2_ (50/10/50 nm), a higher saturation temperature is necessary to remove a water droplet or freeze ice on the TFHs. However, the TFHs fabricated on the SnO_2_/APC/SnO_2_ (10/10/10 nm) electrode rapidly reached 115.2 °C when an input DC voltage of 5.5 V was applied. The higher saturation temperature of TFHs with thinner SnO_2_ layer implies that efficient transduction of electric energy into Joule heating occurred in electrodes with lower sheet resistance^24^. Based on Joule’s law, we can correlate the sheet resistance of transparent electrodes for TFHs and the generated temperature. The power (P) applied to the TFHs during the heating time (t) generated heat (ΔQ_g_)^44, 45^,

2 $${{\rm{\Delta }}{\rm{Q}}}_{{\rm{g}}}=\frac{{{\rm{V}}}^{2}}{{\rm{R}}}{\rm{\Delta }}{\rm{t}}={{\rm{Q}}}_{{\rm{conv}}}={{\rm{h}}}_{{\rm{conv}}}{{\rm{A}}}_{{\rm{conv}}}({{\rm{T}}}_{{\rm{s}}}-{{\rm{T}}}_{{\rm{i}}})$$

3 $${{\rm{T}}}_{{\rm{s}}}=\frac{{{\rm{V}}}^{2}{\rm{\Delta }}{\rm{t}}}{{{\rm{Rh}}}_{{\rm{conv}}}{{\rm{A}}}_{{\rm{conv}}}}+{{\rm{T}}}_{{\rm{i}}}$$

In Equations (2) and (3), Q_conv_ and h_conv_ are a convective heat and heat transfer coefficient, respectively, A_conv_ is the surface area, and T_s_ and T_i_ are the saturation and initial temperatures. Based on Equation (3), it is apparent that the saturation temperature of TFHs increases with increasing input DC voltage (V) and with decreasing resistance (R). Therefore, a lower sheet resistance of a SnO_2_/APC/SnO_2_ electrode is imperative for fabrication of high-performance TFHs with a lower DC input voltage to achieve a temperature of 100 °C.

**Figure 13:**
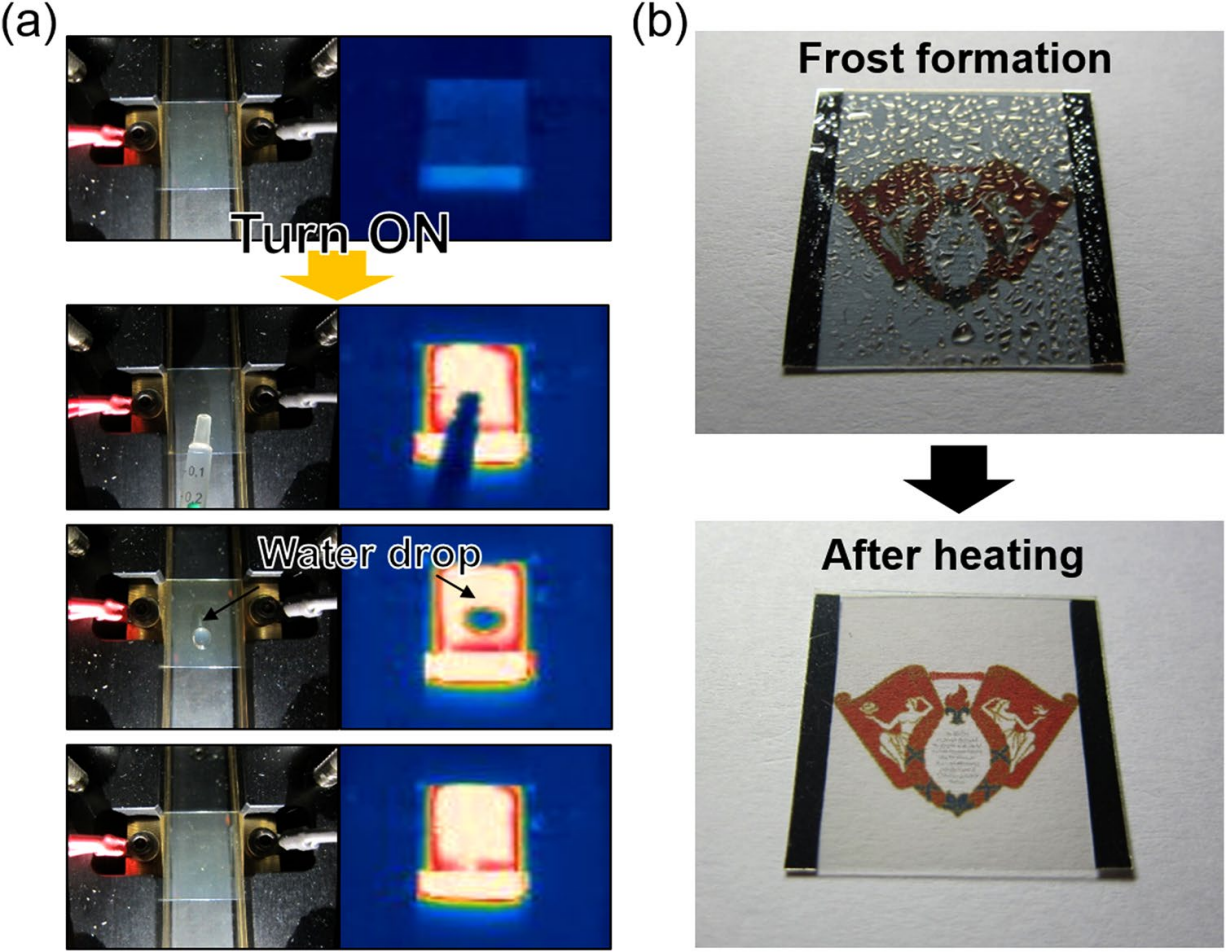
(**a**) Images of the water droplet test on heated TFHs with a SnO_2_/APC/SnO_2_ multilayer electrode. (**b**) Defrosting test results of the SnO_2_/APC/SnO_2_ multilayer film-based TFHs before and after frost formation.

To demonstrate the feasibility of the SnO_2_/APC/SnO_2_ multilayer film-based TFHs, a water droplet test was performed on the heated TFHs. Figure 13a shows the water droplet test images and IR images of the SnO_2_/APC/SnO_2_ (10/10/10 nm)-based TFHs with a saturation temperature of 115 °C. As soon as a DC input voltage of 5.5 V was supplied to the SnO_2_/APC/SnO_2_-based TFHs, a saturation temperature of 115.2 °C was achieved when Joule heating and convection reached a dynamic balance. Therefore, the water droplet disappeared almost immediately due to the high temperature of the SnO_2_/APC/SnO_2_ multilayer film-based TFHs. Figure 13b demonstrates the defrost test of the SnO_2_/APC/SnO_2_-based TFHs before and after frost formation. To ensure uniform frost formation on the surface of the SnO_2_/APC/SnO_2_ electrode, the samples were placed in a refrigerator for 120 min. At an operating voltage of 5.5 V, the frost on the surface of the SnO_2_/APC/SnO_2_ electrode completely disappeared. Effective removal of the frost allowed the Kyung Hee University symbol to appear in the background due to the high transparency of the SnO_2_/APC/SnO_2_ multilayer film. This indicates that the thermally evaporated SnO_2_/APC/SnO_2_ multilayer film is a promising flexible and transparent electrode for a transparent defroster. In addition, it could be employed as a defogging/deicing window for automobiles, helmets, and smart windows due to its flexibility and transparency. In particular, the SnO_2_/APC/SnO_2_-based TFHs could be employed in smart window for automobiles and for transparent buildings because a large-area SnO_2_/APC/SnO_2_ multilayer film could be prepared on a PET substrate using a roll-to-roll based thermal evaporation process. Therefore, a cost-effective defogging/deicing system could be realized on the front or rear windows of an automobile or a large smart window for a transparent building. Furthermore, the SnO_2_/APC/SnO_2_ multilayer film prepared by a thermal evaporation process can be prepared without severe plasma damage which was found in the OMO structure prepared by sputtering. Therefore, the SnO_2_/APC/SnO_2_ electrode could be organic-based electronics or OLEDs as plasma-damage free transparent and flexible electrodes.

## Conclusion

In summary, we investigated the characteristics of themally evaporated SnO_2_/APC/SnO_2_ multilayer films for application as damage-free, indium-free, flexible, and transparent electrodes for high performance flexible and TFHs. The effect of sandwiched SnO_2_ layers or APC interlayer thickness on the resistivity, optical transmittance, and mechanical flexibility of the SnO_2_/APC/SnO_2_ electrodes was investigated to optimize the thickness of each layer. Based on a figure of merit value, we obtained a SnO_2_/APC/SnO_2_ electrode with a low sheet resistance of 9.42 Ohm/square and high optical transmittance of 91.14% as well as smooth surface morphology. In addition, the thermally evaporated SnO_2_/APC/SnO_2_ electrode showed small critical inner/outer bending radius indicating good mechanical flexiblity. The time-dependent temperature profile of TFHs with the SnO_2_/APC/SnO_2_ electrodes demonstrated that the thermally evaporated multilayer electrode is a promising transparent electrode for high performance TFHs. By correlating the sheet resistance of the SnO_2_/APC/SnO_2_ electrode and the performance of TFHs, we showed the importance of a transparent electrode for high performance flexible and transparent TFHs. Consequently, APC inserted into SnO_2_ layer films prepared by thermal evaporation solves the problems of conventional transparent electrode materials and advances transparent electrode technologies for cost-effective TFHs.

## Methods

### Thermal evaporating of SnO_2_/APC/SnO_2_ multilayer film

The flexible and transparent SnO_2_/APC/SnO_2_ multilayer film was continuously evaporated at room temperature on a PET substrate using a thermal evaporation system (NNS Vacuum 15NNS005) without breaking vacuum. At a working pressure of 1 × 10^−6^ Torr in the vacuum chamber, the bottom SnO_2_ layer with various thicknesses was evaporated onto the PET substrate using a SnO_2_ powder source; the operating conditions were an applied voltage of 0.30 V, applied current of 38 A, Z-factor of 0.724, tool factor of 156% and loading plate rotation speed of 10 rpm. After coating the bottom SnO_2_ layer, the metallic APC interlayer with various thicknesses was continuously evaporated onto the bottom SnO_2_ layer at an applied voltage of 0.34 V, applied current of 43 A, Z-factor of 0.529, tool factor of 142% and loading plate rotation speed of 10 rpm. The commercial APC alloy (98 wt% Ag-1 wt% Pd, 1 wt% Cu) was employed as a metal interlayer evaporation source. The thickness of the SnO_2_ and APC layers was precisely controlled by a thickness monitor equipped in the thermal evaporation system. After deposition of the APC interlayer, the top SnO_2_ layer with various thicknesses was finally evaporated on the APC interlayer. The evaporating conditions of the top SnO_2_ layer were the same for the bottom SnO_2_ layer in order to form a symmetric SnO_2_/APC/SnO_2_ structure.

### Characterization of the thermal evaporated SnO_2_/APC/SnO_2_ multilayer film

The electrical and optical properties of the thermal evaporated SnO_2_/APC/SnO_2_ multilayer film were examined using Hall measurements (HL5500PC, Accent Optical Technology) and a UV/visible spectrometer (UV 540, Unicam). The depth-profile of the SnO_2_/APC/SnO_2_ multilayer was examined using X-ray photoelectron spectroscopy (XPS). The work function of the SnO_2_/APC/SnO_2_ multilayer was measured by UPS. The mechanical properties of the SnO_2_/APC/SnO_2_ multilayers film were evaluated using a specially designed inner and outer bending system. The outer bending test induced tensile stress on the film, whereas the inner bending test induced compressive stress. In addition, dynamic fatigue bending and twisting tests were performed using a lab-designed cyclic bending and twist test machine, operated at a frequency of 1 Hz for 2,000 cycles. The resistance of the SnO_2_/APC/SnO_2_ multilayer films was measured throughout cyclic bending.

### Fabrication and evaluations of the TFHs

To demonstrate the feasibility of the SnO_2_/APC/SnO_2_ film as a transparent electrode for TFHs, conventional film heaters (50 × 50 mm^2^) with two-terminal side contacts were fabricated on the thermally evaporated SnO_2_/APC/SnO_2_ multilayer electrode. A 200 nm-thick Ag film was sputtered onto the side region of the SnO_2_/APC/SnO_2_ multilayer to use as a contract electrode. The DC voltage was supplied by a power supply (OPS 3010, ODA technologies) to the SnO_2_/APC/SnO_2_-based TFHs through an Ag contact electrode at the film edge. The temperature of TFHs was measured using a thermocouple mounted on the surfaces of the TFHs and an IR thermal image (A35sc, FLIR).
